# The Importance of Work-Related Events and Changes in Psychological Distress and Life Satisfaction amongst Young Workers in Spain: A Gender Analysis

**DOI:** 10.3390/ijerph17134697

**Published:** 2020-06-30

**Authors:** David Cobos-Sanchiz, María-José Del-Pino-Espejo, Ligia Sánchez-Tovar, M. Pilar Matud

**Affiliations:** 1 Department of Education and Social Psichology, Universidad Pablo de Olavide, 41013 de Sevilla, Spain; 2 Department of Sociology, Universidad Pablo de Olavide, 41013 de Sevilla, Spain; mjpinesp@upo.es; 3 Department of Economics and Social Sciences, Universidad de Carabobo, Maracay 2103, Venezuela; lsanchez@uc.edu.ve; 4 Department of Clinical Psichology, Psichobiology and Methodology, Universidad de La Laguna, 38200 San Cristóbal de La Launa, Spain; pmatud@ull.edu.es

**Keywords:** work-related events, job satisfaction, psychological distress, life satisfaction, gender

## Abstract

A relentless stream of social, technological, and economic changes have impacted the workplace, affecting young people in particular. Such changes can be a major source of stress and can cause a threat to health and well-being. The aim of this paper is to understand the importance of work-related events and changes in the psychological distress and life satisfaction of young workers in Spain. A transversal study was carried out on a sample comprising 509 men and 396 women aged between 26 and 35 years old. The results showed that there were no differences between the men and women in the number of work-related events and changes experienced in the last 12 months, nor in terms of job satisfaction. The results from the multiple regression analysis showed that a greater number of work-related events and changes experienced during the last 12 months were associated with increased psychological distress and reduced life satisfaction amongst men, but this was not the case for women. Although job satisfaction was independent from the men and women’s psychological distress when self-esteem and social support was included in the regression equation, greater job satisfaction was associated with greater life satisfaction for both men and women. It concludes that work-related events and job satisfaction are important for the health and well-being of young people, even though a larger number of work-related events and changes is associated with increased psychological distress and reduced life satisfaction for men only.

## 1. Introduction

Profound social change has been taking place in recent decades in technological and economic terms, having an impact on work and workers. These changes are taking place at increasingly faster speeds and affect most countries across the world. From the late 1980s and, in particular, in the 1990s, researchers supporting different theoretical perspectives have recounted the consequences of the changes in manufacturing systems; the move from an industrial to post-industrial society. These changes go beyond the methods of organizing production, having a clear impact on working conditions and employment opportunities [1]. There is discussion around an environment characterized by volatility, uncertainty, complexity, and ambiguity (VUCA) where companies, in response to almost unpredictable social expectations, undergo constant and rapid change [2]. Among the effects of globalization, it is worth highlighting that economic, social, and health crises are no longer confined to just one country, but rather they spread to other countries—sometimes more quickly than others.

Within this context, working life has experienced significant changes, caused by an ever more global and flexible system [3]; these are changes that are contributing to a significant loss of work, with an increase in unemployment, underemployment, and precarious employment throughout the world [4]. According to the International Labor Organization (ILO), the main problem in the world’s labor markets is poor quality employment. Millions of people are compelled to accept poor working conditions. Recent data show that, in 2018, the majority of the total global working population of 3.3 billion did not have an adequate level of economic security, material well-being, and equal opportunities [5]. This tends to affect young people most of all, as individuals that are extremely vulnerable to an ever-changing environment, where situations of abnormal employment, temporary employment, part-time work, outsourcing, individual contracts, and self-employment prevail. The changes in the world of work that young people are facing, whether due to the introduction of new technologies or the type of employment in itself, take place in such a way that young people often do not have the chance to adapt to the position [6].

For millions of young people in the European Union, finding a job is extremely difficult. In some Southern European countries, more than half of all young adults are unemployed, a situation that was made even worse by the last financial crisis. This entails problems of a psychosocial nature, but can also have devastating consequences for the countries concerned—and the European Union itself [7] in a post-Brexit context, due to the social tensions that can arise from unemployment. In Spain, in particular, youth unemployment rates double those of the adult population and affect more than half of the population [8]. The most relevant aspect has been the change in the industrial trend of male employment: stable jobs for life have made way for constant change with periods of unemployment, instability, and precariousness throughout one’s working life. In addition to the difficulty of entering the labor market for the first time, replicated across Europe, the problems arising from the Spanish economy based on unstable productive industries must also be considered [1].

It could be assumed that this dynamic reflects the stamina of young people, but in reality it shows an overwhelming situation that hinders them in their daily routine, characterized by an uncontrolled flow of new demands that constantly force them to search for new jobs, which consistently fail to meet their expectations due to their temporary nature. According to Vendramin [9], switching jobs is part of a “normalized” journey of employment instability for young people. These changes are involuntary for 33.7% of women and 22% of men. The reality of young workers has been put in the spotlight by different visions. According to data from the ILO [5], women are more likely to take casual work, and just like young people, they are prone to establishing weak ties with the employment market.

It is undeniable that working is fundamental for the economic and psychological well-being of an individual, and of society in general [4]. In current day society, a person’s job is one of their most important sources of identity and it plays a vital economic and social role for the majority of people [10]. However, employment and working conditions can involve factors that are not people-oriented, and this affects their well-being. The links between work and health have become a central issue in organizational literature, and workers are becoming more and more aware of the importance of health in their work–life balance [11,12].

In terms of young workers, it is particularly important to understand that their initiation into the world of work is taking place under adverse circumstances; due to the lack of jobs currently on offer on the market and also due to the lack of appreciation for their personal conditions due to inexperience [6]. Although work can be a source of stress, it is a fundamental aspect of life, as working age adults spend the majority of their waking hours at work [13]. Work is particularly important for a young person’s personal development, and due to the restricted possibilities of entering the labor market, they can end up accepting precarious employment with unfavorable conditions that put their physical and mental integrity at risk.

It is worth nothing that, despite the employment crisis and the aforementioned changes in production systems, the vision and expectations of young Spanish people with regard to work and professional life continue to be largely similar to those of previous generations. For them, work is a fundamental part of their lives, whether it is more instrumental and utilitarian (young people from an industrial background or from peripheral rural and semi-rural areas), or more self-fulfilling (young urbanites) [14].

In this respect, despite the fact that, among the realistic expectations of young Spanish people is just how difficult it is to gain a foothold in the labor market, this does not mitigate a major psychological and social impact, which can trigger severe and significant consequences for individuals that endure over time [15]. Such circumstances may be, in fact, an important source of stress that can alter the physiology and mental well-being of individuals [16]. Work-related events and changes thus pose a threat to the health and well-being of women and men alike.

In recent decades ample research has been carried out on the psychosocial risks of work, such as stress in the workplace, and a link was found between psychosocial risks and their consequences on physical, mental, and social health [17]. In accordance with data from the Quebec National Institute for Public Health [18], there are several scientific studies that have shown that the presence of one or more psychosocial risks in the workplace can impinge upon workers’ mental and physical health, increasing the risk of accidents. It was also stressed that workers with a low level of education working in precarious employment have to face increased psychosocial risks at work, which affects their health and hinders the possibility of improved life conditions. Likewise, it was highlighted that men and women are not exposed to the same psychosocial risks in the workplace, but that women tend to be more exposed.

It is important to understand that when assessing work-related psychosocial factors, analyzing job satisfaction is also important. Although there is no sole definition, it is believed that job satisfaction reflects a pleasant emotional state where people value their work or work experience positively [19]. Job satisfaction is a global concept that covers several aspects and is determined by specific work elements, such as the workplace or the job’s characteristics, by specific personal factors such as skill or psychological status, and by non-specific factors such as demographic, cultural, and community aspects [19,20]. There is evidence that job satisfaction has an impact on individual performance and company results, as well as affecting the health and lifestyle quality of the worker. For example, it has been found that job satisfaction is associated with trust in the company [21], work performance [22], and self-efficacy [23], whilst workers that are unsatisfied can see their mental and physical health suffer due to mood changes or psychosomatic complaints, reduced efficiency, more time off, and more requests for a change in role [24].

Symptoms of depression and anxiety have been named collectively as psychological distress [25], although other symptoms such as somatic issues or insomnia are also included within psychological distress [26,27,28]. Both clinically and in research, psychological distress is a commonly used indicator for mental health and psychopathologies [27], as well as being associated with physical health. There is evidence showing that psychological distress is associated with higher mortality rates for various reasons [29], and with several inflammatory markers [30]. It has also been found to increase the risk of diseases such as arthritis, cardiovascular disease, and chronic obstructive pulmonary disorder [25].

Many of these effects have been studied less in young people than they have been in adults, perhaps due to morbidity and mortality rates being comparatively lower in the former group. It should be noted, however, that various health issues onset at a young age, which may affect the individual throughout the rest of their life [31].

Self-esteem and social support stand out amongst the psychosocial factors related to health and well-being [32,33,34,35]. Self-concept refers to describing and evaluating oneself, including one’s psychological and physical characteristics, qualities, skills, and roles. Self-esteem is the degree to which such qualities and characteristics are perceived as being positive [36]. There are many factors that determine a person’s self-esteem, including individual values, attitudes, wishes, family issues, and social factors related to work and the type of work [37]. There is evidence that self-esteem has consequences on central aspects of life and high self-esteem leads to good mental and physical health, satisfaction with close relationships, and social support [38,39], as well as being associated with job performance [37] and professional prestige and income [10].

Across all societies, gender is fundamental in organizing work, and work is fundamental for socially constructing gender. Although there is empirical evidence that men and women are similar in the majority of their psychological features [40,41], most societies believe that differences remain, and that they should take on different roles; and they are treated differently depending on the gender assigned to them at birth. Gender is a social construct [42] that restricts people and gives them different roles and positions. Traditional gender roles consider women as carers and men as the backbone of the family [43], assuming that working is vital for a man’s mental health, but somewhat secondary for women, whilst the opposite assumption occurs within family roles [44]. These assumptions are not supported by the empirical evidence that shows that the quality of job positions is associated with less psychological distress amongst men and women [44,45], and that the similarities between men and women are clearer than the differences, amongst a series of factors that are important for the family–work association [46].

Despite this, it is still believed that the commitment of a woman to work is lower than that of a man, and the classification of gender is an important deciding factor when it comes to professional interests [47]. Although there has been a trend towards more equal gender roles in recent decades [48], gender stereotypes that present significant differences between men and women in terms of their features, occupations, and behavior still exist [49,50]. In spite of a woman’s role in the workplace having become more widespread in recent years in many countries [51] total working equality has not yet been achieved, with salary gaps and job segregation remaining [51,52,53]. An example of this is that men still dominate the more prestigious and creative roles, as well as the technical positions [53].

Although the importance of research in the psychological aspects of work has received more recognition in recent decades, and research has been done on the psychosocial risks inherent to the workplace and the working environment [17], the impact on health due to stress at work understood as work-related events and changes has been studied less. Furthermore, the positive aspects of work such as job satisfaction or the presence of personal resources such as self-esteem, and social resources such as social support, are rarely considered in these studies. These are all variables that can differ from men to women and can be important in determining health and well-being.

The aim of this paper is therefore to understand the importance of work-related events and changes experienced in the last year in psychological distress and life satisfaction for young people in Spain, including satisfaction with the job role, self-esteem, and emotional and instrumental social support in the prediction model, all of which will be assessed by analyzing men and women separately.

The hypotheses are:

**Hypothesis** **1.** *Men and women who have experienced a greater number of work-related events and changes, and who report low job satisfaction, low self-esteem, and low social support will also report greater psychological distress*.

**Hypothesis** **2.** *Men and women who have experienced a lesser number of work-related events and changes, and who report high job satisfaction, high self-esteem, and high social support will also report greater life satisfaction*.

## 2. Materials and Methods

### 2.1. Participants

The sample consisted of 509 men and 396 women aged between 26 and 35 years old. The average age of the men was 30.13 (SD = 2.69) and for women was 30.08 (SD = 2.81), the difference was not statistically significant, t(903) = 0.28, *p* = 0.78. Their professions were different: 37% was non-manual labor, 34.9% was manual labor, and 28.1% had professions that required university studies. There were also differences in their level of education, even though it was most common for them to have studied at university (41.8%), 33.7% had secondary school education and 24.5% had only basic education. More than half of the sample (59.3%) was single, 39% was married or in a civil partnership, and 1.7% was separated or divorced.

### 2.2. Measures

#### 2.2.1. Dependent Variables: Psychological Distress and Life Satisfaction

Psychological distress was assessed using scales of somatic symptoms, anxiety and insomnia, and severe depression as per the GHQ-28 [54], each of which includes 7 items that gathers information on general health over recent weeks. Example items are “Been feeling nervous and strung-up all the time?”, “Felt constantly under strain?”, “Felt that life isn’t worth living?”, and “Felt that life is entirely hopeless?”. The Likert scale was used, allocating weightings from 0 (no symptoms) to 3 (greater discomfort). The internal consistency in the sample group of this paper for the 21 items is 0.92.

Life satisfaction was assessed with the Satisfaction with Life Scale (SWLS) [55]. It is made up of 5 items with a Likert-style 7-point response scale ranging from 1 (completely disagree) to 7 (completely agree). It is a tool that has been used in many countries, Spain included, and has shown suitable psychometric properties for men and women [35,56]. The internal consistency in the sample group of this paper was 0.84.

#### 2.2.2. Independent Variables: Number of Work-Related Events and Changes Experienced in the Last 12 Months, Job Satisfaction, Self-Esteem, and Social Support

The work-related events and changes were assessed using four items where participants were asked whether in the last 12 months they had experienced the following: (1) change of employment, (2) loss of employment, (3) starting new employment; and (4) change in employment conditions. Each item was scored with a 0 if the person had not experienced it in the previous 12 months and with 1 if they had. The total score for work-related events and changes was obtained by adding together the responses from the 4 items, so the score range is between 0 (for the complete absence of work-related events and changes) and 4, which is the maximum score.

Job satisfaction was assessed using the job satisfaction questionnaire [57]. It is an open response test where there are 5 questions about whether the person is satisfied in their job, if it is the job they chose, if they want a change, and to what extent they feel fulfilled. The responses to each of the questions were scored quantitatively by applying a code created and approved by Matud [57]. The internal consistency for the sample group in this paper was 0.76.

Self-esteem was assessed using the Spanish version of the York Self-Esteem Inventory [58], a questionnaire made up of 51 items that takes an overall measurement of self-esteem, reflecting the assessment of several skills including personal, interpersonal, family, achievement, physical attractiveness, and the assessment of the degree of uncertainty in themselves. The answer format is on a 4-point scale that ranges from “never” (scored with a 0) to “always”, which is scored with a 3. With this sample group in this paper the internal consistency was 0.94.

Social support was assessed using the social support scale [59]. It is made up of 12 items, answers to which are given on a 4-point Likert scale that ranges from 0 (never) to 3 (always), and they assess the social support perceived emotionally (7 items) and instrumentally (5 items). The internal consistency of the sample in this paper was 0.84 for emotional social support and 0.80 for instrumental social support.

Furthermore, each participant was given a sociodemographic and employment data collection sheet.

### 2.3. Procedure

Participants were volunteers and were not paid for their participation in this study. The sample was chosen through various work centers of Spanish companies all over Spain, from all production sectors. To collect data, the social networks of psychology and sociology students at 7 Spanish universities were analyzed, who were trained for the testing step and received course credits for this task. After verbal informed consent was received, the questionnaires were completed individually on paper by people that met the following criteria: (1) aged between 16 and 35 years old; (2) with work experience and either working (work experience means having, or having had, a formal employment contract) or not; and (3) able to understand and speak Spanish.

This study is part of broader research on the importance of personal and social factors in men and women’s well-being, and it was assessed positively by the Animal Research and Well-being Ethics Committee at the University of La Laguna (study approval no. 2012-0040).

### 2.4. Statistical Analysis

Descriptive analyses were carried out to understand the socio-demographic characteristics of the participants. The reliability of the internal consistency of the study factors was calculated using Cronbach’s alpha coefficient. The comparisons between men and women were calculated using the Student’s *t*-test. The bivariate associations between variables were calculated using Pearson’s r correlation coefficient except for the educational level where Spearman’s Rho was used as it is an ordinal variable with 7 levels, from 0 (for no studies) to 6 (for university studies spanning 6 years). To test the hypotheses and determine the importance of the number of work-related events and changes, job satisfaction, self-esteem and social support in psychological distress, and life satisfaction amongst men and women, hierarchical multiple regression analyses were made. The age and level of studies were incorporated at the first step (Model 1) to control their effect. At step 2 (Model 2), the number of work-related events and changes and job satisfaction. Finally, at step 3 (Model 3), self-esteem and emotional and instrumental social support were incorporated. The correlations and the multiple regression analyses were made independently for the sample of women and the sample of men. The statistical analyses were performed using the IBM SPSS Statistics for Windows software, version 22.0 (IBM Corp., Armonk, N.Y., USA).

## 3. Results

**Figure 1:**
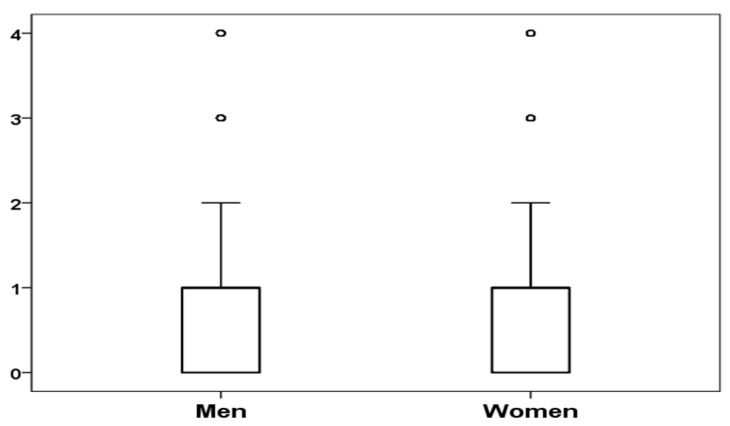
Boxplot of number of work-related events and changes experienced in the previous 12 months by men and women.

Figure 1 shows the boxplot of the number of work-related events and changes experienced in the previous 12 months. No statistically significant differences were found between men and women in terms of the number of work-related events and changes experienced in the previous 12 months, with an average of 1.04 for men (SD *=* 1.18) and 0.94 (SD = 1.15) for women, t(903) = 1.26, *p* = 0.21.

**Figure 2:**
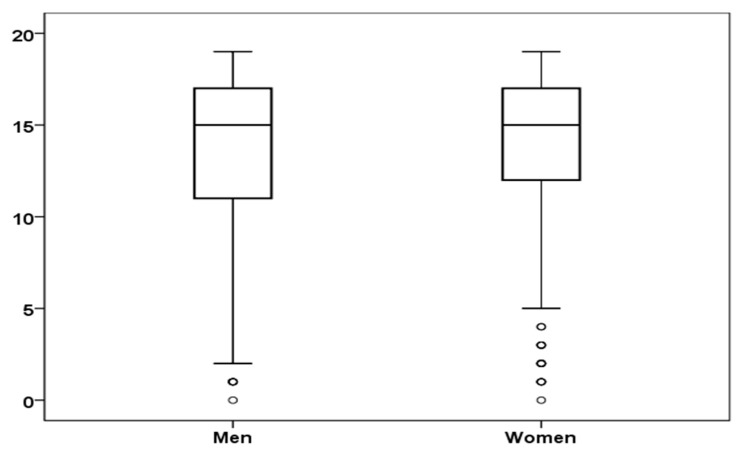
Boxplot of job satisfaction scores for men and women.

Figure 2 shows the boxplot of job satisfaction scores for men and women. For men, the average for job satisfaction was 13.55 (SD *=* 4.43) and for women 14.05 (SD *=* 4.27), which were not statistically significant differences either, t(903) = −1.62, *p* = 0.11.

In Table 1 are the correlation coefficients between the age, level of studies, number of work-related events and changes, job satisfaction, self-esteem and social support with the psychological distress, and life satisfaction amongst men and women. As it is possible to observe, age is independent from the psychological distress and life satisfaction amongst men and women, and although for men said variables are also independent from the level of studies, women with a higher level of studies have less psychological distress and greater life satisfaction. For both men and women, a higher number of work-related events and changes are associated with increased psychological distress and less life satisfaction, whilst more job satisfaction, self-esteem, and social support are associated with more life satisfaction and less psychological distress.

Table 2 shows the main results from the hierarchical multiple regression analysis in which the dependent variable was psychological distress for the male sample, with Table 3 showing the female sample. As it is possible to observe, Model 1 was only statistically significant in the female sample, albeit with the only statistically significant predictor being the level of studies (β = −0.17, *p* < 0.01). Including the number of work-related events and changes and job satisfaction in Model 2 produced a statistically significant increase in R^2^, with the Beta weights being statistically significant for both variables amongst men but only job satisfaction amongst women. Including self-esteem and emotional and instrumental social support in Model 3 also produced a statistically significant increase in R^2^, although for the female sample only self-esteem was statistically significant (β = −0.50, *p* < 0.001), whilst in the male sample self-esteem (β = −0.49, *p* < −001) and instrumental social support (β = −0.12, *p* < 0.05) was. Model 3, with all the independent variables in the equation, predicted 28% of the variability in psychological distress amongst men and 31% of psychological distress amongst women. For the male sample, psychological distress was associated with lower self-esteem, a higher number of work-related events and changes in the past year, and less instrumental social support whilst for the females it was only associated with a lower self-esteem.

Table 4 shows the main results from the hierarchical multiple regression analysis in which the dependent variable was life satisfaction for the male sample, with Table 5 showing the female sample. As it is possible to observe, Model 1 was only statistically significant in the female sample where a higher level of study was associated to greater job satisfaction. Including the number of work-related events and changes that had taken place in the last year and job satisfaction in Model 2 produced a statistically significant increase in R^2^ for men and women, with the Beta weights being statistically significant for both variables, and showing that greater life satisfaction was associated to greater job satisfaction and a lower number of work-related events and changes in the previous year. Including self-esteem and emotional and instrumental social support in Model 3 produced a statistically significant increase in R^2^ for men and women, with the Beta weights for self-esteem and emotional social support in the male sample and self-esteem and instrumental social support in the female sample being statistically significant.

Model 3, with all the regression variables, predicted 27% of the variance in life satisfaction for men and 31% for women. For men, increased life satisfaction was associated with increased job satisfaction, greater emotional social support, higher self-esteem, and less work-related events and changes during the last year. For women, increased life satisfaction was associated to higher self-esteem, more job satisfaction, and greater instrumental social support.

## 4. Discussion

The aim of this paper was to understand the importance of work-related events and changes experienced in the last year in assessing psychological distress and life satisfaction for male and female young workers in Spain, including these factors in the prediction model together with the number of work-related events and changes, and job satisfaction. Self-esteem and emotional and instrumental social support were also included in the regression equation, with the aim of understanding the relative weight that social, personal, and work factors have on psychological distress and life satisfaction. A hierarchical regression model was used, and men and women were analyzed separately, given the evidence that gender is an important distinction in the workplace.

It highlights that, in regression analysis, work-related events and changes experienced in the previous year and job satisfaction were statistically significant for men, but for women, only job satisfaction, was statistically significant. This coincides with what has been reported in literature [44,45]; for both men and women, the quality of job positions is associated with less psychological distress and better health. The fact that only job satisfaction was statistically significant for women could be a reflection of women facing situations of inequality, segregation, imbalances, and gender stereotypes in the labor market [49,50], which is still happening regardless of the academic level reached by this group in recent years.

It is well known that gender places young men and women unequally in both the education and the labor market [60]. There is extensive literature on the gender gap, in general and in the Spanish labor market in particular, which looks at employment discrimination, its evolution throughout the life cycle and, specifically, pay discrimination [61,62,63,64]. Employers still hold stereotypes about women’s productivity and, in general, tend to regard women as being less committed to paid work than men [65,66]. This reality is reported in studies that reveal the distribution of roles in accordance with gender in workplaces [51,52,53], aspects, which are often highlighted in female working environments.

With regard to the predictors of psychological distress and life satisfaction, there were some significant differences between men and women. In both groups, age was independent from psychological distress and life satisfaction, as was the case with the level of studies for men. For women, a higher level of studies was associated with less psychological distress and greater life satisfaction, despite the small size of the effect and its greatly reduced statistical significance when self-esteem and social support were included in the regression equation.

The first hypothesis, proposing that men and women who have experienced a greater number of work-related events or changes, and who report low job satisfaction, low self-esteem, and low social support would also report greater psychological distress, was only partially supported. Although in the male sample a larger number of work-related events and changes taking place in the last year and less job satisfaction predicted psychological distress (Model 2), when self-esteem and social support (Model 3) were included in the regression equation, job satisfaction ceased to be statistically significant in predicting psychological distress. For women, although in Model 2 less job satisfaction predicted increased psychological distress, job satisfaction ceased to be statistically significant in predicting psychological distress when self-esteem and social support (Model 3) were included in the regression equation. Self-esteem ended up being an important predictor of psychological distress for the male sample and the only predictor for the females. These results coincide with those of other studies [33,38] and confirm the importance of self-esteem on psychological well-being for both men and women. These results force us to consider the value of self-esteem and psychological well-being as health contributors, as highlighted by some authors in studies on psychological distress and the workplace [32,33,34,35]. The results highlight the lack of importance of social support in predicting psychological distress, as it was only statistically significant in the male group, despite literature reporting social support as a protective factor of psychological distress.

The second hypothesis, which proposed that men and women who have experienced a lesser number of work-related events and changes, and who report high job satisfaction, high self-esteem, and high social support would also report greater life satisfaction, was also only partially supported. In fact, in the male group, greater life satisfaction was associated with greater job satisfaction, increased emotional social support, higher self-esteem, and less work-related events and changes. However, for women, in the final model, when self-esteem and social support were incorporated, the number of work-related events and changes ceased to be statistically significant, and the social support, which was associated in a statistically significant way with greater life satisfaction, was instrumental and not emotional. This suggests that self-esteem and social support are valuable factors when surveying disrupting situations in life satisfaction for both groups. It should be noted that the perceived social support in particular has been considered by several authors as an element that facilitates protection against situations that create psychological distress.

The results found highlight that there are differences between men and women in the predictive value of work-related events and changes in psychological distress, with job events and changes being much more associated with psychological distress in young men rather than women. This could perhaps be a consequence of traditional social practices and gender stereotypes that further underscore working roles amongst men more so than women [23,47,49,50], and therefore work-related events and changes could equate to a bigger threat for men’s mental health than for women’s. In any case, it also highlights that there were no differences between men and women in terms of their job satisfaction, and this was important for predicting life satisfaction for both sexes.

## 5. Conclusions

The results allow us to broaden our knowledge about the relevance of work-related events and changes on the health and well-being of women and men. In this respect, the findings of our research serve as a basis for further studies aimed at the in-depth research into distress in young workers, including looking into factors that implicate the work environment as a potential trigger of psychological distress. In particular, the difference between men and women in the predictive importance that work-related events and changes have in psychological distress and life satisfaction (a construct that refers to the feeling of well-being with oneself in one’s surroundings) has been highlighted, with this being much greater amongst young men than young women.

In conclusion, work-related events and changes and job satisfaction are important for the health and well-being of young workers, even though a larger number of work-related events and changes amongst only men are associated with greater psychological distress and reduced life satisfaction. It is important to highlight that, for young workers, life satisfaction, social support, and self-esteem were shown to be important factors to considered in research in relation to the psychological distress created by adverse circumstances in the working environment.

## 6. Limitations of the Study and Future Areas of Research

The study has some limitations and it should be noted that a convenience sample was used, so there can be no guarantee that this is representative of young Spanish people. Moreover, the study is transversal, so we cannot speak of cause–effect relationships. In addition, the percentage of variance explained in psychological distress and in life satisfaction does not exceed 31%. Certain aspects could have been studied in greater depth, and remain open to subsequent study in greater detail. In particular, it would be interesting to use the Holmes-Rahe Life Stress Scale, a psychological scale used to measure susceptibility to stress-induced health problems, as well as to introduce the locus of control as a study variable. Finally, there should be some mention of the recent COVID-19 pandemic. This study was carried out using the data of young Spanish people collected in the last year. Obviously, conditions have changed since the data were used, in a dynamic and changing context. We understand that the main results are still valid in this context, however, it is reasonable to consider highly likely that the socio-economic situation may be aggravated in light of the current situation, which may have an impact on the psychosocial occupational risks to which young people are exposed.

## Figures and Tables

**Table 1:** Correlations between the dependent and independent variables amongst men and women.

	Men		Women	
	Psychological Distress	Life Satisfaction	Psychological Distress	Life Satisfaction
Age	−0.08	0.04	0.06	0.02
Level of studies ^$^	−0.05	0.09	−0.17 **	0.16 **
No. of work-related events and changes	0.16 ***	−0.12 **	0.10 *	−0.14 **
Job satisfaction	−0.13 **	0.31 ***	−0.20 ***	0.35 ***
Self-esteem	−0.52 ***	0.36 ***	−0.55 ***	0.45 ***
Emotional social support	−0.18 ***	0.36 ***	−0.25 ***	0.34 ***
Instrumental social support	−0.20 ***	0.29 ***	−0.23 ***	0.36 ***

*Note:* * *p* < 0.05; ** *p* < 0.01; *** *p* < 0.001. ^$^ = Spearman’s *Rho* correlation coefficient.

**Table 2:** Summary of the hierarchical multiple regression analysis with psychological distress as a dependent variable for the male sample.

	Model 1		Model 2		Model 3	
	β	t-Value	β	t-Value	β	t-Value
Age	−0.07	−1.66	−0.05	−1.08	−0.03	−0.67
Level of studies	−0.06	−1.28	−0.04	−0.92	−0.02	−0.39
No. of work-related events and changes			0.15	3.44 **	0.12	3.16 **
Job satisfaction			−0.12	−2.73 **	−0.05	−1.26
Self-esteem					−0.49	−12.20 ***
Emotional social support					0.05	1.02
Instrumental social support					−0.12	−2.29 *
R^2^	0.01		0.05		0.29	
Adjusted R^2^	0.01		0.04		0.28	
R^2^Change	0.01		0.04		0.25	
ANOVA (*F*-value, df)	2.29 (2, 506)		6.07 (4, 504) ***		29.85 (7, 501) ***	

*Note:* β = Standardized regression coefficient. * *p* < 0.05; ** *p* < 0.01; *** *p* < 0.001.

**Table 3:** Summary of the hierarchical multiple regression analysis with psychological distress as a dependent variable for the female sample.

	Model 1		Model 2		Model 3	
	β	t-Value	β	t-Value	β	t-Value
Age	0.03	0.55	0.04	0.84	0.05	1.08
Level of studies	−0.17	−3.38 **	−0.13	−2.58 *	−0.07	−1.63
No. of work-related events and changes			0.09	1.77	0.03	0.69
job satisfaction			−0.17	−3.34 **	−0.06	−1.34
Self-esteem					−0.50	−11.02 ***
Emotional social support					−0.09	−1.42
Instrumental social support					−0.01	−0.10
R^2^	0.03		0.07		0.33	
Adjusted R^2^	0.03		0.06		0.31	
R^2^Change	0.03		0.04		0.26	
ANOVA (*F*-value, df)	6.32 (2, 393) **		7.01 (4, 391) ***		26.72 (7, 388) ***	

*Note:* β = Standardized regression coefficient. * *p* < 0.05; ** *p* < 0.01; *** *p* < 0.001.

**Table 4:** Summary of the hierarchical multiple regression analysis with life satisfaction as a dependent variable for the male sample.

	Model 1		Model 2		Model 3	
	β	*t*-Value	β	*t*-Value	β	*t*-Value
Age	0.04	0.80	−0.01	−0.13	−0.00	−0.09
Level of studies	0.09	1.94	0.06	1.49	0.05	1.21
No. of work-related events and changes			−0.11	−2.55 *	−0.09	−2.31 *
Job satisfaction			0.31	7.26 ***	0.25	6.52 ***
Self-esteem					0.24	5.86 ***
Emotional social support					0.25	4.62 ***
Instrumental social support					0.03	0.47
R^2^	0.01		0.11		0.28	
Adjusted R^2^	0.01		0.11		0.27	
R^2^Change	0.01		0.10		0.16	
ANOVA (*F*-value, df)	2.26 (2, 506)		16.19 (4, 504) ***		27.34 (7, 501) ***	

*Note:* β = Standardized regression coefficient. * *p* < 0.05; ** *p* < 0.01; *** *p* < 0.001.

**Table 5:** Summary of the hierarchical multiple regression analysis with life satisfaction as a dependent variable for the female sample.

	Model 1		Model 2		Model 3	
	β	*t*-Value	β	*t*-Value	β	*t*-Value
Age	0.04	0.87	0.02	0.43	0.02	0.42
Level of studies	0.17	3.37 **	0.10	1.99 *	0.03	0.73
No. of work-related events and changes			−0.11	−2.41 *	−0.08	−1.83
Job satisfaction			0.32	6.79 ***	0.24	5.42 ***
Self-esteem					0.31	6.80 ***
Emotional social support					0.10	1.55
Instrumental social support					0.16	2.54 *
R^2^	0.03		0.15		0.33	
Adjusted R^2^	0.02		0.14		0.31	
R^2^Change	0.03		0.12		0.18	
ANOVA (*F*-value, df)	5.74 (2, 393) **		16.72 (4, 391) ***		26.72 (7, 388) ***	

*Note:* β = Standardized regression coefficient. * *p* < 0.05; ** *p* < 0.01; *** *p* < 0.001.
